# Hospital in the Nursing Home program reduces emergency department presentations and hospital admissions from residential aged care facilities in Queensland, Australia: a quasi-experimental study

**DOI:** 10.1186/s12913-016-1275-z

**Published:** 2016-02-09

**Authors:** Lijun Fan, Xiang-Yu Hou, Jingzhou Zhao, Jiandong Sun, Kaeleen Dingle, Rhonda Purtill, Sam Tapp, Bill Lukin

**Affiliations:** 1 School of Public Health and Social Work, Queensland University of Technology, Victoria Park Road, Kelvin Grove, Brisbane, QLD 4059 Australia; 2 Bureau of Investment Promotion, Wuwei City, 733000 Gansu Province P. R. China; 3 Queensland Health, Brisbane, QLD 4029 Australia; 4 Department of Emergency Medicine, Royal Brisbane and Women’s Hospital, Brisbane, QLD 4006 Australia

**Keywords:** Emergency department, Hospital in the Nursing Home, Health services for the aged

## Abstract

**Background:**

There has been considerable publicity regarding population ageing and hospital emergency department (ED) overcrowding. Our study aims to investigate impact of one intervention piloted in Queensland Australia, the Hospital in the Nursing Home (HiNH) program, on reducing ED and hospital attendances from residential aged care facilities (RACFs).

**Methods:**

A quasi-experimental study was conducted at an intervention hospital undertaking the program and a control hospital with normal practice. Routine Queensland health information system data were extracted for analysis.

**Results:**

Significant reductions in the number of ED presentations per 1000 RACF beds (rate ratio (95 % CI): 0.78 (0.67–0.92); *p* = 0.002), number of hospital admissions per 1000 RACF beds (0.62 (0.50–0.76); *p* < 0.0001), and number of hospital admissions per 100 ED presentations (0.61 (0.43–0.85); *p* = 0.004) were noticed in the experimental hospital after the intervention; while there were no significant differences between intervention and control hospitals before the intervention. Pre-test and post-test comparison in the intervention hospital also presented significant decreases in ED presentation rate (0.75 (0.65–0.86); *p* < 0.0001) and hospital admission rate per RACF bed (0.66 (0.54–0.79); *p* < 0.0001), and a non-significant reduction in hospital admission rate per ED presentation (0.82 (0.61–1.11); *p* = 0.196).

**Conclusions:**

Hospital in the Nursing Home program could be effective in reducing ED presentations and hospital admissions from RACF residents. Implementation of the program across a variety of settings is preferred to fully assess the ongoing benefits for patients and any possible cost-savings.

## Background

In Australia, over 160,000 people live permanently in residential aged care facilities (RACFs), and this increased by 25 % over the past decade [1]. The vast majority of residents (96 %) are aged 65 and over, and 70 % require high level care [1]. People residing in RACFs represent one of the most vulnerable groups in society, characterized by multiple chronic problems susceptible to acute exacerbations and secondary comorbidities [2]. Therefore residents have disproportionately high demands for acute medical services, and they frequently present to emergency departments (EDs) and many require hospital admission from ED [2–4]. However their visits are often unnecessary owing to their relatively minor and repeated health problems, or serious chronic disease without appropriate end-of-life care plans. Unnecessary ED presentations and hospital admissions by RACF residents put further pressure on already overtaxed services, potentially further exacerbating ED and hospital overcrowding and compromising access to timely and high-quality acute care [5, 6]. Unnecessary hospital attendances can simultaneously expose residents to potential complications, such as hospital-acquired infections, falls, and disorientation, and decrease their quality of life [7].

Pilot hospital EDs in Queensland, Australia are committed to encouraging a coordinated intervention incorporating multiple individual strategies to reduce unnecessary ED and hospital attendances from RACF residents and to provide equitable, timely and safe clinical care for residents, entitled “Hospital in the Nursing Home (HiNH)” program. This program is funded by Queensland State Government and involves a responsive and dynamic team of ED-based nurses working in partnership and coordinating with RACF staff and other health providers. Hospital admission and ED presentation avoidance programme, such as HiNH, is a key policy aimed at early interventions with RACF residents and nursing staff, to reduce the incidence of unnecessary attendances to EDs and hospitals; and this is considered vital for maintaining an effective and responsive acute care system [4, 8].

In the present study, we aim to investigate the impact of this HiNH program on ED presentations and hospital admissions from RACFs.

## Methods

### Study design, setting and population

A quasi-experimental study design was used. This research design included an experimental group and a control group, and each group was analysed for both pre-test and post-test performance. Random assignment to the experimental or control group was not possible, as it was an intervention design applied in natural settings.

Our study included a three-month pre-test period from 1 June 2005 to 31 August 2005, and a 12-month post-test period commencing on 1 March 2011. It was undertaken in two Queensland hospitals and the RACFs in each of these two hospitals’ catchment areas, with one hospital receiving the intervention (Royal Brisbane and Women’s Hospital (RBWH)) and the other acting as a control group (Logan Hospital (LH)). The intervention group has implemented the HiNH intervention since February 2006. The control group received normal practice and was not exposed to any similar or comparable interventions.

Patients were eligible for inclusion in the study population if they had presented to the ED of RBWH or LH over the three-month pre-test period and the 12-month post-test period and they were residents of RACFs.

### Intervention

The Hospital in the Nursing Home (HiNH) intervention aims to ensure that the patient receives correct treatment in the correct location at correct times. Four fundamental components of the HiNH intervention are:

i. HiNH allocates clinical staff to manage aged care residents with actual or potential acute symptoms in the RACF, which would otherwise require admission to either an ED or hospital.
ii. HiNH provides support and education for RACF staff and general practitioners (GPs) to improve their ability to provide acute medical care for residents.
iii. HiNH seeks senior medical decision-making at an early stage of presentation to ED, and has a key contact person (an ED nurse who has previous geriatric care experience as well) that facilitates open discussion and communication between families, RACF staff, GPs and hospital staff, in order to enable efficient movement of patients within the institutions and disease management for them.
iv. HiNH coordinates the discharge of RACF residents from EDs and inpatient units. Where returning to RACF is applicable or end-of-life care in RACFs is desirable, the HiNH team would liaise with patients’ GP and support for RACF staff to continue with care that otherwise would have been provided in hospital.

### Outcome measures

The primary outcomes of this study were:

i. The number of ED presentations per 1000 RACF beds per month (i.e., ED presentation rate). ED presentation is defined as an episode that “occurs following the arrival of the patient at the emergency department and is the earliest occasion of being registered clinically or triaged” [9].
ii. The number of hospital admissions via ED per 1000 RACF beds per month (i.e., hospital admission rate per RACF bed). Hospital admission refers to “a formal process, and follows a medical officer making a decision that a patient needs to be admitted for appropriate management or treatment of their condition, or for appropriate care or assessment of their needs. Admitted patient services are either provided on a same-day basis or involve a stay in hospital overnight or longer” [10].
iii. The number of hospital admissions per 100 ED presentations per month (i.e., hospital admission rate per ED presentation).

### Data collection

Data for this study were sourced from two computerized patient tracking systems used by Queensland public hospitals, i.e., Emergency Department Information System (EDIS) and Hospital Based Corporate Information System (HBCIS). All RACF residents that presented to the ED of RBWH and LH during June-August 2005 and March 2011-February 2012 were identified in EDIS, and variables that described patient demographics and ED presentation-related characteristics were extracted, including: unique unit record (UR) number, age, gender, Australasian Triage Scale (ATS) score, ICD (International Classification of Diseases) code for ED primary diagnosis, date and time of arrival, date and time of actual departure, and departure status. Hospital admission data was then sourced from HBCIS records for any of the RACF residents admitted to hospital from the ED, during the period of June-August 2005 and March 2011-February 2012. The UR numbers were used to retrieve a wide range of data related to the hospital admission, including: patient demographics, date and time of admission, date and time of discharge, diagnosis code, and discharge type. Data were collected on two groups of RACF residents over the investigated periods, people who had ED presentations and people who were admitted to hospital after first presenting to the ED. The total number of funded RACF beds in the catchment areas of RBWH and LH was also collected from consultations with the HiNH program director.

### Data analysis

Pearson chi-square test was used to compare between the pre-intervention period and the post-intervention period about attending patients’ demographics and clinical characteristics. Log-linear model was applied to investigate whether the ED presentation rate (and hospital admission rate per RACF bed) among RACF residents was significantly different between the intervention and control hospitals and between the pre-test period and post-test period. To compare the difference in hospital admission rate per ED presentation, binary logistic regression was conducted to model hospital admission as a function of different groups that the ED patients were in (i.e., intervention or control group, and pre-test or post-test group), after controlling for a set of confounders. Data were analysed using SPSS v. 21 (IBM Corp., Armonk, NY). Statistical significance was set at *p* < 0.05.

### Ethics approval

Our study was approved by the institutional Human Research Ethics Committee (ethics clearance number: 1000000457). Ethics approval was obtained from the Royal Brisbane and Women’s Hospital and the Logan Hospital as well.

## Results

**Fig. 1:**
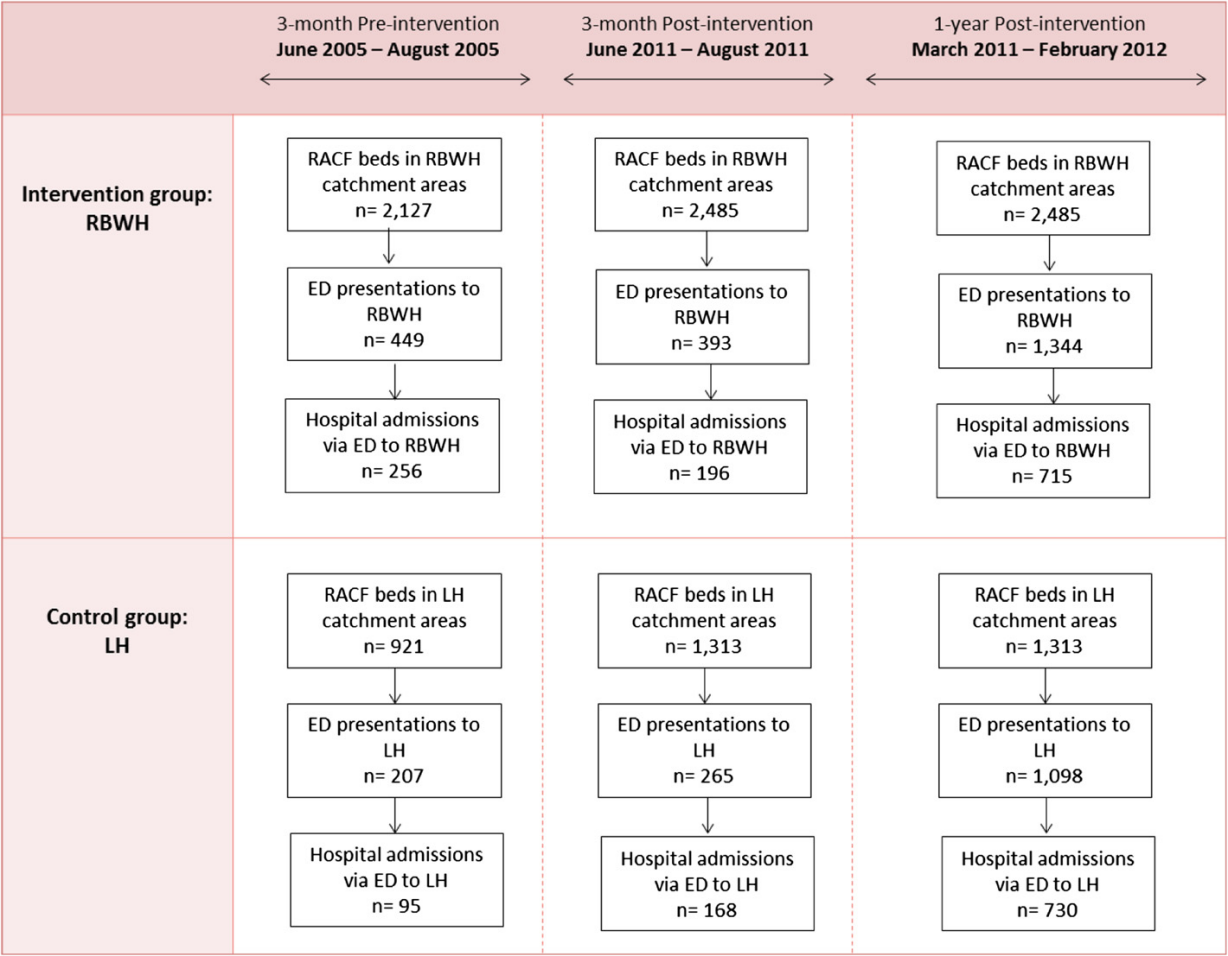
Overview of ED presentations and hospital admissions via ED from RACFs to the intervention (RBWH) and control hospital (LH) during pre- and post-test period

Figure 1 summarises the numbers of ED presentations and hospital admissions via the ED from RACFs to the two hospitals over time. In total, there were 449 ED presentations and 256 hospital admissions to RBWH from 2127 RACF beds in the three-month pre-intervention period (from June to August 2005), while were 393 ED presentations and 196 hospital admissions from 2485 RACF beds in the three-month post-intervention period from June to August 2011. For the examined whole one-year post-intervention period from March 2011 to February 2012, the number of ED presentations and hospital admissions was 1344 and 715, respectively. In contrast, for LH, the number of ED presentations and hospital admissions was 207 and 95 for the three-month pre-intervention period from its 921 RACF beds, 265 and 168 for the three-month post-intervention period from its 1313 RACF beds, and 1098 and 730 for the whole one-year post-intervention period from 1313 RACF beds.

Patients’ demographics, and their ED presentation and hospital admission related characteristics were compared between the three-month pre-intervention period and the corresponding three-month post-intervention period within RBWH (Table 1). As the results showed, between the pre-intervention and post-intervention periods, there were no significant differences in the distribution of patient age, gender, ATS, attendance day and time, while there was some difference in patients’ ED primary diagnosis for attending acute hospitals. This was similar for patients that were admitted to the inpatient units via ED.

**Table 1:** Comparison of RBWH patients’ demographic and clinical characteristics in the pre- and post-test period

Indicators	ED presentation to RBWH			Hospital admission to RBWH		
	3-month Pre-test	3-month Post-test	*p*-value	3-month Pre-test	3-month Post-test	*p*-value
	(*n* = 449)	(*n* = 393)		(*n* = 256)	(*n* = 196)	
Age Group, n (%)			0.997			0.913
< 65	29 (6.5)	26 (6.6)		9 (3.5)	10 (5.1)	
65–74	58 (12.9)	49 (12.5)		32 (12.5)	21 (10.7)	
75–84	137 (30.5)	123 (31.3)		81 (31.6)	63 (32.1)	
85–94	193 (43.0)	169 (43.0)		113 (44.1)	86 (43.9)	
≥ 95	32 (7.1)	26 (6.6)		21 (8.2)	16 (8.2)	
Gender, n (%)			0.887			0.239
Male	168 (37.4)	145 (36.9)		88 (34.4)	78 (39.8)	
Female	281 (62.6)	248 (63.1)		168 (65.6)	118 (60.2)	
Australasian Triage Scale^a^, n (%)			0.480			0.225
ATS 1&2 (seen immediately)	80 (17.8)	78 (19.8)		57 (22.3)	54 (27.6)	
ATS 3–5 (can wait)	369 (82.2)	315 (80.2)		199 (77.7)	142 (72.4)	
Attendance Day, n (%)			1.000			0.467
Weekday	339 (75.5)	296 (75.3)		184 (71.9)	134 (68.4)	
Weekend	110 (24.5)	97 (24.7)		72 (28.1)	62 (31.6)	
Attendance Time, n (%)			0.105			0.687
Working hours	284 (63.3)	227 (57.8)		86 (33.6)	62 (31.6)	
After hours	165 (36.7)	166 (42.2)		170 (66.4)	134 (68.4)	
Primary Diagnosis, n (%)			0.003^**^			0.007^**^
Injury & Poisoning	104 (23.2)	108 (27.5)		59 (23.0)	40 (20.4)	
Respiratory	51 (11.4)	39 (9.9)		36 (14.1)	27 (13.8)	
Circulatory	42 (9.4)	31 (7.9)		28 (10.9)	20 (10.2)	
Digestive	40 (8.9)	14 (3.6)		27 (10.5)	6 (3.1)	
Genitourinary	22 (4.9)	29 (7.4)		12 (4.7)	21 (10.7)	
Musculoskeletal & Skin	38 (8.5)	18 (4.6)		20 (7.8)	9 (4.6)	
Mental & Neurological	23 (5.1)	17 (4.3)		6 (2.3)	9 (4.6)	
Other	129 (28.7)	137 (34.9)		68 (26.6)	64 (32.7)	

* *p* < 0.05, ** *p* < 0.01

^a^ATS 1&2: patients who must be seen immediately; ATS 3-5: Patients who can wait and will be seen in order of arrival

### Results on ED presentation rate

Intervention-control comparison and pre-post comparison of ED presentation rate were conducted. Results were presented in Table 2.

**Table 2:** Comparison of three outcome measures between the intervention (RBWH) and control group (LH) and between the pre- and post-test period

ED presentation rate (Number of ED presentations per 1000 RACF beds):					
	3-month pre-test (Mean ± SD)	3-month post-test (Mean ± SD)	1-year post-test (Mean ± SD)	Rate Ratio (95 % CI)	*p*-value
	06/2005–08/2005	06/2011–08/2011	03/2011–02/2012	3-month post-test vs. 3-month pre-test	
Intervention group	70.37 ± 6.59	52.72 ± 1.45	45.07 ± 5.84	0.75 (0.65, 0.86)	<0.0001^**^
Control group	74.92 ± 12.52	67.28 ± 3.83	69.69 ± 7.92	0.90 (0.75, 1.08)	0.246
Rate Ratio (95 % CI) Intervention vs. Control	0.94 (0.80, 1.11)	0.78 (0.67, 0.92)	0.65 (0.60, 0.70)		
*p*-value	0.455	0.002^**^	<0.0001^**^		
Hospital admission rate per RACF bed (Number of hospital admissions per 1000 RACF beds):					
	3-month pre-test (Mean ± SD)	3-month post-test (Mean ± SD)	1-year post-test (Mean ± SD)	Rate Ratio (95 % CI)	*p*-value
	06/2005–08/2005	06/2011–08/2011	03/2011–02/2012	3-month post-test vs. 3-month pre-test	
Intervention group	40.12 ± 8.74	26.29 ± 0.84	23.98 ± 3.37	0.66 (0.54, 0.79)	<0.0001^**^
Control group	34.38 ± 5.13	42.65 ± 6.23	46.33 ± 6.20	1.24 (0.96, 1.60)	0.093
Rate Ratio (95 % CI) Intervention vs. Control	1.17 (0.92, 1.48)	0.62 (0.50, 0.76)	0.52 (0.47, 0.57)		
*p*-value	0.199	<0.0001^**^	<0.0001^**^		
Hospital admission rate per ED presentation (Number of hospital admissions per 100 ED presentations):					
	3-month pre-test (Mean ± SD)	3-month post-test (Mean ± SD)	1-year post-test (Mean ± SD)	Odds Ratio^a^ (95 % CI)	*p*-value
	06/2005–08/2005	06/2011–08/2011	03/2011–02/2012	3-month post-test vs. 3-month pre-test	
Intervention group	56.63 ± 8.46	49.91 ± 2.58	53.34 ± 5.16	0.82 (0.61, 1.11)	0.196
Control group	46.18 ± 4.58	63.20 ± 5.85	66.60 ± 6.34	2.10 (1.36, 3.26)	0.001^**^
Odds Ratio^a^ (95 % CI) Intervention vs. Control	1.29 (0.87, 1.93)	0.61 (0.43, 0.85)	0.59 (0.49, 0.70)		
*p*-value	0.209	0.004^**^	<0.0001^**^		

* *p* < 0.05, ** *p* < 0.01; *SD* standard deviation, *CI* confidence interval

^a^ Odds ratio was obtained using binary logistic regression that modelled impact of the HiNH intervention and impact of the test period, respectively, on the outcome, after controlling for patients’ age, gender, Australasian Triage Scale (ATS), primary clinical diagnosis, season, day (weekday/weekend) and time (working hours/after hours) of patients’ ED departure

During the pre-intervention period, ED presentation rate per month in the two hospitals was not significantly different (rate ratio: 0.94; 95 % CI: 0.80–1.11; *p* = 0.455). While over the post-intervention period, the ED presentation rate in RBWH was significantly lower than that in LH (the corresponding three-month post-test: rate ratio: 0.78; 95 % CI: 0.67–0.92; *p* = 0.002; one-year post-test: rate ratio: 0.65; 95 % CI: 0.60–0.70; *p* < 0.0001). Compared with the pre-test period, there was no significant change in the number of ED presentations from RACFs in the control hospital (post-test vs. pre-test: rate ratio: 0.90; 95 % CI: 0.75–1.08; *p* = 0.246); while there was a significant reduction in ED presentations in the experimental hospital after the intervention (rate ratio: 0.75; 95 % CI: 0.65–0.86; *p* < 0.0001). After adjusting for the differences between pre-test vs. post-test groups and intervention vs. control groups, there was a 17 % reduction in the number of ED presentations from RACFs in the intervention hospital during the intervention period.

### Results on hospital admission rate per RACF bed

As illustrated by Table 2, during the pre-intervention period, RBWH dealt with a higher hospital admission rate per RACF bed than LH, but the magnitude of difference between them was not significant (rate ratio: 1.17; 95 % CI: 0.92–1.48; *p* = 0.199). By contrast, after the intervention, RBWH had a significantly lower admission rate per RACF bed than LH (three-month post-test: rate ratio: 0.62; 95 % CI: 0.50–0.76; *p* < 0.0001; one-year post-test: rate ratio: 0.52; 95 % CI: 0.47–0.57; *p* < 0.0001). Furthermore, compared with the pre-test period, hospital admission rate per RACF bed in the control hospital increased in the post-test period (rate ratio: 1.24; 95 % CI: 0.96–1.60; *p* = 0.093), while on the contrary, admission rate in the intervention hospital decreased to a significant degree (rate ratio: 0.66; 95 % CI: 0.54–0.79; *p* < 0.0001). Considering the pretest-posttest and the intervention-control inherent differences, the number of hospital admissions decreased by 47 % after the intervention.

### Results on hospital admission rate per ED presentation

Table 2 demonstrates the results of comparison of hospital admission rate per ED presentation, after adjusting for patients’ age, gender, ATS, primary diagnosis, season, day and time of their ED departure. Over the pre-intervention period, on average across the three-month periods, hospital admission rate per ED presentation was not significantly different between RBWH and LH (rate ratio: 1.29; 95 % CI: 0.87–1.93; *p* = 0.209). While over the post-test period, the hospital admission rate per ED presentation in RBWH was lower than that in LH (three-month post-test: rate ratio: 0.61; 95 % CI: 0.43–0.85; *p* = 0.004; one-year post-test: rate ratio: 0.59; 95 % CI: 0.49–0.70; *p* < 0.0001). Hospital admission rate per ED presentation significantly increased in the control hospital in the post-test period (rate ratio: 2.10; 95 % CI: 1.36–3.26; *p* = 0.001); while it showed a decrease in the intervention hospital, although not significantly (rate ratio: 0.82; 95 % CI: 0.61–1.11; *p* = 0.196). There was a 36 % reduction in the adjusted hospital admission rate per ED presentation with the intervention.

## Discussion

This study, to our knowledge, represents the first effort to quantitatively evaluate effectiveness of the Hospital in the Nursing Home (HiNH) program, on its impact on the ED and hospital occupancy among RACF patients. We comparatively analysed three outcomes of interest (ED presentation rate, hospital admission rate per RACF bed, and hospital admission rate per ED presentation) between the intervention hospital (RBWH) and control hospital (LH), showing non-significant differences between hospitals during the pre-intervention period but substantially significant decreases in three outcomes in RBWH after the intervention. The further pre-test and post-test comparison of three outcomes within each hospital also supports reductions for RBWH. The pre-post ED presentation rate and hospital admission rate per RACF bed fall significantly; and the hospital admission rate per ED presentation, though not significantly, shows a reduction with the intervention as well. We speculate the reason why this reduction on hospital admission rate per ED presentation was non-significant might be due to a general trend of growing demand for hospital admissions over time, considering that the control group, on the contrary, had an increased number of hospital admissions per ED presentation in the post-test period. Hence, this study has reasonable data to support that the HiNH intervention is associated with reduced numbers of ED presentations and hospital admissions from RACF residents.

Results of our study are consistent with previous findings that alternate programs could reduce ED presentations and hospital admissions from RACF patients [11–13]. Other studies, however, reported non-significant or no reductions on patient attendances to acute hospital [14–16]. Differences in the details of interventions were likely to be associated with success or failure of interventions. Those studies looked at the impact of provision of enhanced primary care or additional RACF care from multidisciplinary geriatric team or geriatric nurse practitioners; while our study provides the first evidence to investigate the effect of an ED nurse led intervention incorporating altered care within RACF and enhanced care management from both primary care and hospital care providers. Despite variations in intervention effects across studies, we consider that hospitals and RACFs in different locations continuously have aligned incentives to work out appropriate intervention components for reducing the elderly’s acute hospital visits, owing to their devotion to care for the distinct needs and improve comfort of RACF patients in particular and desire to relieve the common overcrowding in acute settings. Further outcomes associated with the care-home model, such as hospital readmission and mortality rates, were evaluated in a previous Italian study, although not examined by our study [17]. It found a Discharge Planning relying on a Care-Home model did not significantly change the readmission rates and mortality rates as compared with routine care, however, it would reduce mortality rates when it was followed by a long-term care plan [17]. This may indicate the importance of management of the continuity of care, which was also represented by one of the goals of the HiNH intervention.

There is good evidence to support the possible reductions in ED presentations and hospital admissions from RACFs with the intervention. Researchers have demonstrated that there is scope for avoiding a sizeable proportion of unnecessary ED presentations and hospital admissions from RACF [12, 18–20]. The proportion of potentially avoidable ED presentations or hospital admissions varies nationally and internationally between 7 % and 48 %, according to previous studies [7, 18, 19, 21–26]. Our study has estimated a 17 % reduction in ED presentation from RACF residents, a 47 % reduction in hospital admission per RACF bed, and a 36 % reduction in hospital admission per ED presentation, which all sit within the range previously reported.

These avoidable presentations and admissions might be achieved with a range of simple interventions [12, 18–20]. For instance, some hospital care could be alternatively provided on site in RACFs, including urinary catheter change, administering intravenous antibiotics, wound care, PEG tube, etc., which is among the practices of our studied intervention. Early intervention or additional acute care at RACFs reduces residents’ need to attend hospitals for extended care; and as a result, patients will probably have improved clinical outcomes and quality of care [2, 3, 12, 27, 28]. We assume so because the frail RACF residents are often prone to adverse events associated with ED and hospital attendances, and the hospital environment itself is unsuited for the special and complex needs of elderly RACF residents. RACF patients commonly require residential care focusing on caring and supporting, while the emphasis of ED services is curing and treating and allows little time for adequate assessment of RACF patients’ complex needs due to their distinguished characteristics (chronic illness, cognitive impairments and physical dependency).

However, factors such as lack of resources for RACFs (e.g., RACF staffing, necessary equipment or supplies, primary care), lack of confidence in care that can be provided within RACF, inadequate care planning and communication, bureaucratic and legal concerns, and conflicting stakeholder preferences, are believed to be associated with failures in avoiding unnecessary attendances to EDs and hospitals [2, 12, 20]. We hypothesize that effectiveness of the HiNH program in reducing ED presentations and hospital admissions are very likely owing to the components of this program that have addressed some of the above identified barriers. The HiNH team provides clinical support and general education to RACF staff, such that RACF staff are more qualified to manage an extended range of their own residents’ simple acute symptoms alternatively within the facility. While registered nurses in RACFs feel lacking in confidence to provide certain care that actually they could, they have the chance to ask feedbacks from equal colleagues (i.e., the HiNH team). Often the HiNH team serves as a core contact person who links partnership and promotes communication among family members, RACF staff, GPs, ED staff, ward staff, and treating team, which, to the largest extent, avoids conflicting messages delivered and increases knowledge of each patient (e.g., what the patient’s unique needs are and whether the patient has any advanced care directives or preference for palliative care). In particular, ED nurses employed in HiNH have previous working experience in aged care as well; and this makes them better understand RACF patients’ real needs than ED and hospital-based staff who usually care for general population. The HiNH team manages to understand each patient’s situation, recognises their personhood, and maintains their dignity.

Our study shows that the HiNH intervention is not associated with significant changes in terms of patients’ characteristics (age, gender, ATS), and day and time of their ED presentations and hospital admissions. We had expected that after the intervention, there would be a higher proportion (not the number) of patients presenting to ED or being admitted to hospitals during the after hours (as the intervention operates during working hours only). We speculate that the reason for our finding of non-significant difference in attendance time (working hours/after hours) after the intervention might be related with the HiNH program functioning beyond its operational hours. It is very likely that during the after hours when HiNH is not operating, if there is any non-urgent patient otherwise requiring ED presentation, would still be kept by RACF staff in the facility because of awareness that the HiNH staff will provide appropriate care for them on the following day. This finding is congruent with that of another study [12].

This study has limitations as well. First, within the limit of this study design, it is essentially impossible to state definitely that whether it is the intervention only that leads to the changes in outcome measures, considering the complex contextual realities. Second, there is general rule that RACF residents in the catchment area of a certain hospital ED will all be sent to that hospital for treatment. However, in practice, patients would sometimes cross boundaries and be diverted to other local EDs when that hospital is busy. This phenomenon would contaminate our results on effectiveness. Realizing this issue and looking at available evidence, we found that in 2011, RBWH had only 14 bypass hours (0 % of total hours) but LH had 1995 bypass hours (23 % of total hours). Effect of this on hospitals was probably a net flow of ambulance patients (and thus RACF patients) away from LH to other hospitals, and a net flow of patients toward RBWH. This meant the size of effectiveness we obtained from this study was probably reduced rather than magnified, but we are unable to know the exact difference. Third, it is practically unable for this study to analyse the role of different RACFs in a multilevel analysis. However, all RACFs in the catchment areas of the intervention and control hospitals were federal funded, and their performances were all assessed against a set of legislated Accreditation Standards uniformly adopted in Queensland Australia. The composition of RACFs in the two areas was also similar, with comparable percentage of high-care RACF beds and low-care RACF beds. The lack of major differences in the organizational model of the RACFs in the two areas may suggest that different RACFs have little effect on the results of our study.

## Conclusions

Our study suggests the Hospital in the Nursing Home (HiNH) program could be effective in reducing emergency department (ED) presentations and hospital admissions from patients in residential aged care facilities (RACFs). This is somewhat similar to the effect of “created capacity” for EDs and acute hospitals, because a meaningful number of ED and inpatient beds can thus be emptied to serve more other patients in urgent need. The RACF patients also benefit, as they could be cared at right places and right times and could avoid unnecessary traumatic hospital visits. This HiNH program looks valued in this study. Implementation of the program across a variety of settings is preferred to fully assess the ongoing benefits for patients and any possible cost savings.

## Abbreviations

ATS: Australasian triage scale
CI: confidence interval
ED: emergency department
EDIS: Emergency Department Information System
GP: general practitioner
HBCIS: Hospital Based Corporate Information System
HiNH: Hospital in the Nursing Home
ICD: international classification of diseases
LH: Logan Hospital
RACF: residential aged care facilities
RBWH: Royal Brisbane and Women’s Hospital
SD: standard deviation
